# Supplementary material for: Integrated proteomics reveals autophagy landscape and an autophagy receptor controlling PKA-RI complex homeostasis in neurons
Source: Nat Commun. 2024 Apr 10;15:3113. doi: 10.1038/s41467-024-47440-z (PMC11006854; doi:10.1038/s41467-024-47440-z)
Supplement: Supplementary file 1 — Supplementary Information [file 41467_2024_47440_MOESM1_ESM.pdf]

Integrated proteomics reveals autophagy landscape and an autophagy receptor controlling PKA-RI complex homeostasis in neuron

Supplementary Fig. 1

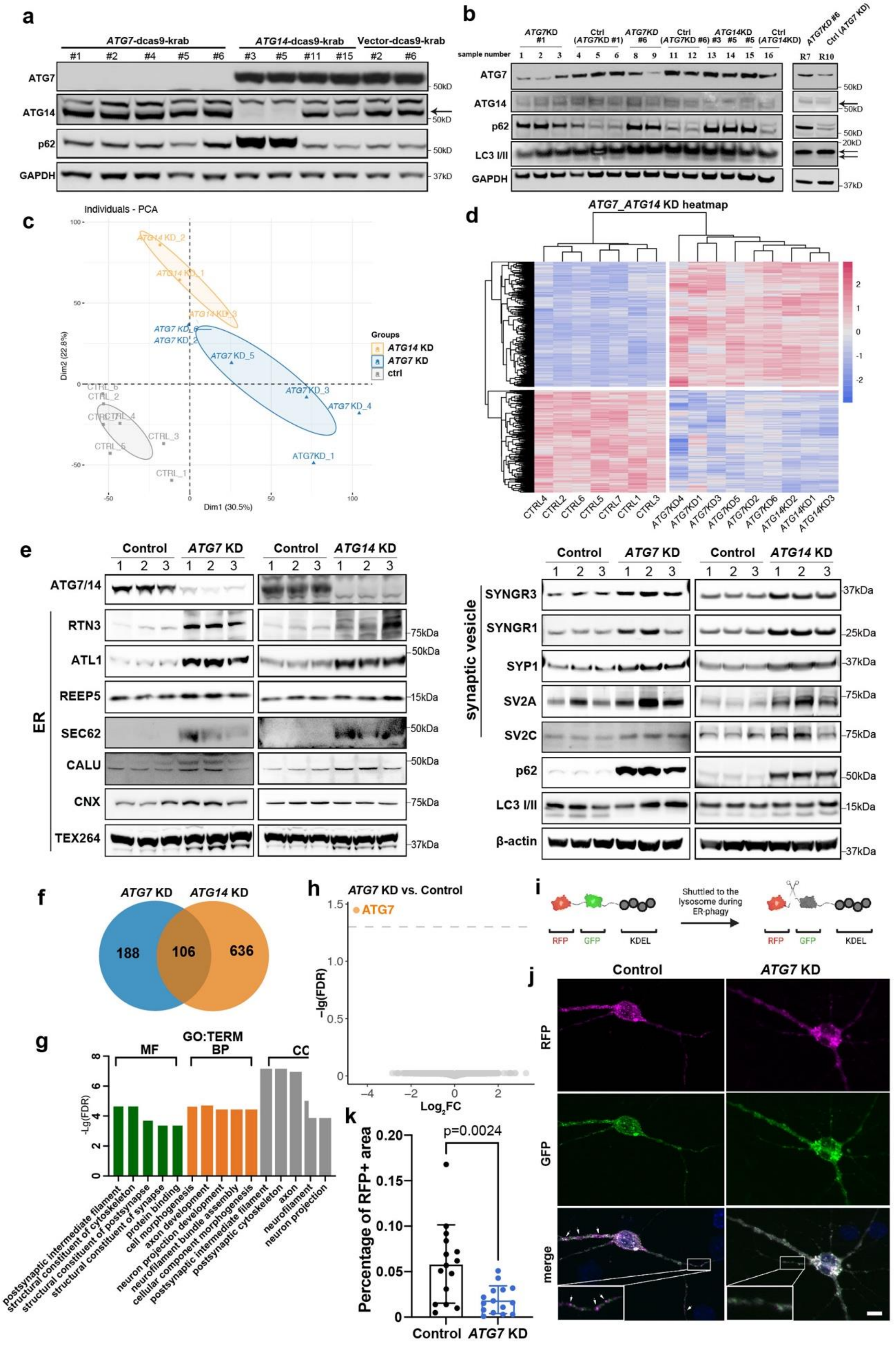
a
ATG7-dcas9-krab
ATG14-dcas9-krab
Vector-dcas9-krab
ATG7
ATG14
p62
GAPDH
b
ATG7KD #1
Ctrl (ATG7KD #1)
ATG7KD #6
Ctrl (ATG7KD #6)
ATG14KD
Ctrl (ATG14KD)
sample number
LC3 I/II
c
Individuals - PCA
Dim1 (30.5%)
Dim2 (22.8%)
Groups
ATG14 KD
ATG7 KD
ctrl
d
ATG7_ATG14 KD heatmap
e
Control
ATG7 KD
ATG14 KD
ATG7/14
ER
RTN3
ATL1
REEP5
SEC62
CALU
CNX
TEX264
synaptic vesicle
SYNGR3
SYNGR1
SYP1
SV2A
SV2C
p62
LC3 I/II
β-actin
f
ATG7 KD
ATG14 KD
188
106
636
g
GO:TERM
MF
BP
CC
-Lg(FDR)
postsynaptic intermediate filament
structural constituent of cytoskeleton
structural constituent of postsynapse
structural constituent of synapse
protein binding
cell morphogenesis
axon development
neuron projection development
neurofilament bundle assembly
cellular component morphogenesis
postsynaptic intermediate filament
postsynaptic cytoskeleton
axon
neurofilament
neuron projection
h
ATG7 KD vs. Control
ATG7
-lg(FDR)
Log2FC
i
Shuttled to the lysosome during ER-phagy
RFP
GFP
KDEL
j
Control
ATG7 KD
RFP
GFP
merge
k
p=0.0024
Percentage of RFP+ area
Control
ATG7 KD

Supplementary Fig. 1. Generation and proteomic analysis of human *ATG7* or *ATG14* KD iNeurons

(a) Immunoblot analysis of multiple clones of *ATG7* KD, *ATG14* KD, or control with antibodies as indicated. Human mutant iNeurons generated from subclones *ATG7* KD#1 and #6, *ATG14* KD#3 and #5, Vector #2 and #6 were selected for proteomics analysis.

(b) Immunoblot analysis of indicated iNeuron clone samples submitted for proteomic analysis. Detailed clone information for each sample is in Supplementary table S1a.

(c) Principal component analysis (PCA) of 6 *ATG7* KD iNeurons (blue), 3 *ATG14* KD iNeurons (orange), and 7 Control iNeurons (gray) for proteomics from Figure S1b.

(d) Heatmap analysis of Log_2FC of DEPs (FDR<0.05) for human iN proteomics samples.

(e) Immunoblot analysis of ER (left) or SV (right) proteins as indicated in control, *ATG7* KD and *ATG14* KD human iNeurons.

(f) Venn diagram showing the overlap between the downregulated DEPs of *ATG7* KD (blue) and of *ATG14* KD (orange) iNeurons ($p< 0.05$, $log_2FC< - 2$ SD).

(g) GO enrichment analysis of 106 downregulated DEPs shared between *ATG7* KD and *ATG14* KD iNeurons from (e). MF: Molecular Function, BP: Biological Process, CC: Cellular Component.

(h) Volcano plot of the DEGs from transcriptomic data of three batches of *ATG7* KD iNeurons vs. Control iNeurons. Orange dots represent the only DEG identified (FDR< 0.05). Dashed line is at -Lg (FDR)= 1.3.

(i) Schematic of the ER-phagy reporter RFP-GFP-KDEL. The GFP signal is quenched when fused with lysosomes to yield the RFP only signal.

(j-k) Immunofluorescence images (i) of Control and *ATG7* KD iNeurons transfected with ER-phagy reporter plasmids after nutrient starvation (glucose and sodium pyruvate starvation for 48 h and quantification of the percentage of RFP only area (j). Inset, magnified images of the boxed region. Arrows indicated RFP-only puncta in the processes. Scale bar, 10 μm. Two-sided unpaired *t*-test from 3 independent biological replicates, n=15. Data are shown as mean ± SEM.

Supplementary Fig. 2

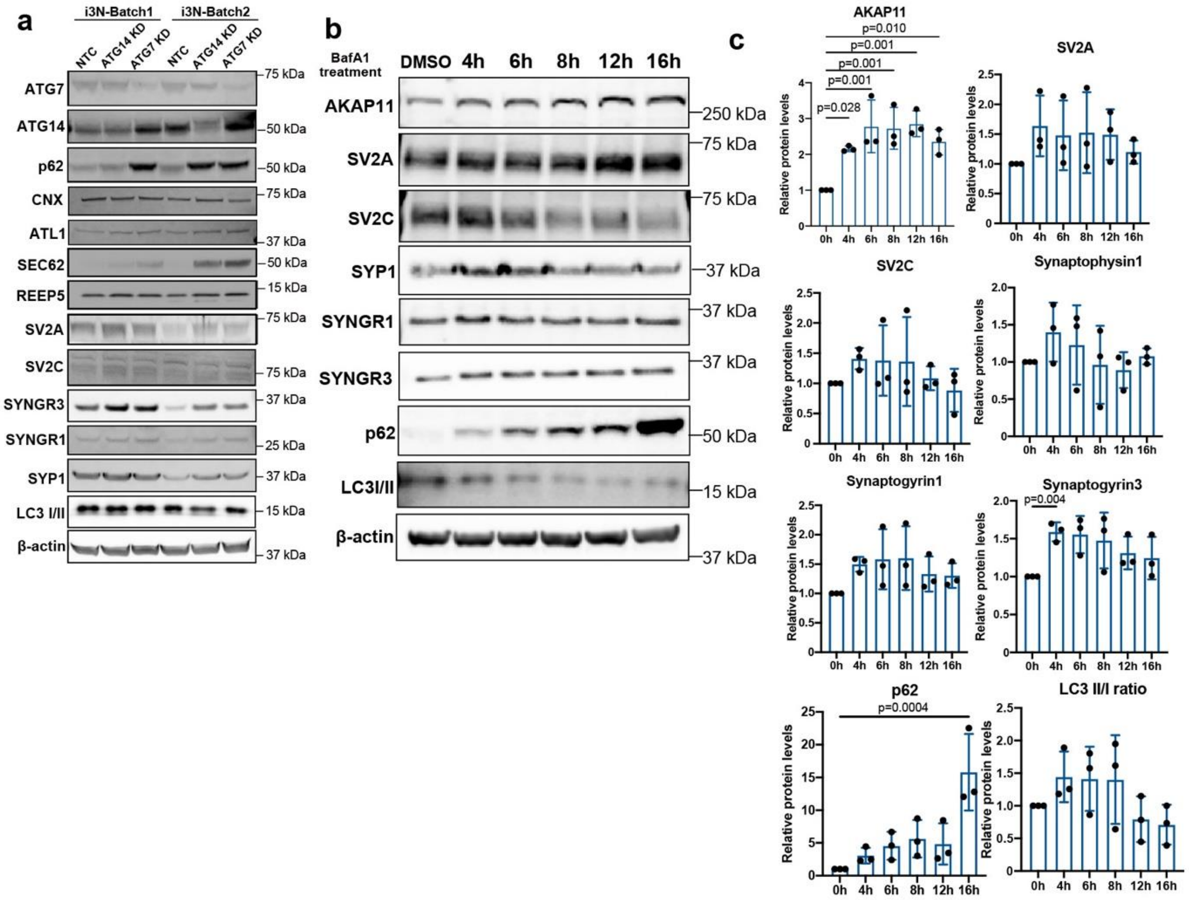

Supplementary Fig. 2. Analysis of ER and SV related proteins in multiple iNeuron lines

(a) Immunoblot analysis of ER and SV proteins as indicated in control, *ATG7* KD, and *ATG14* KD iNeurons derived from the inducible iPSC cell lines (i3N) from two independent biological replicates.

(b and c) Immunoblot analysis of ER and SV proteins as indicated in WT iNeurons upon a time-dependent (4h, 6h, 8h, 12h, 16h) treatment of Bafilomycin A1(100nM). DMSO-treated iNeurons serve as a negative control. Relative protein levels were normalized to the loading control β-actin (c). Data were collected from 3 biologically independent replicates. One-way ANOVA, Dunnett's multiple comparisons test for comparison between groups. All data are shown as mean ± SEM.

Supplementary Fig. 3

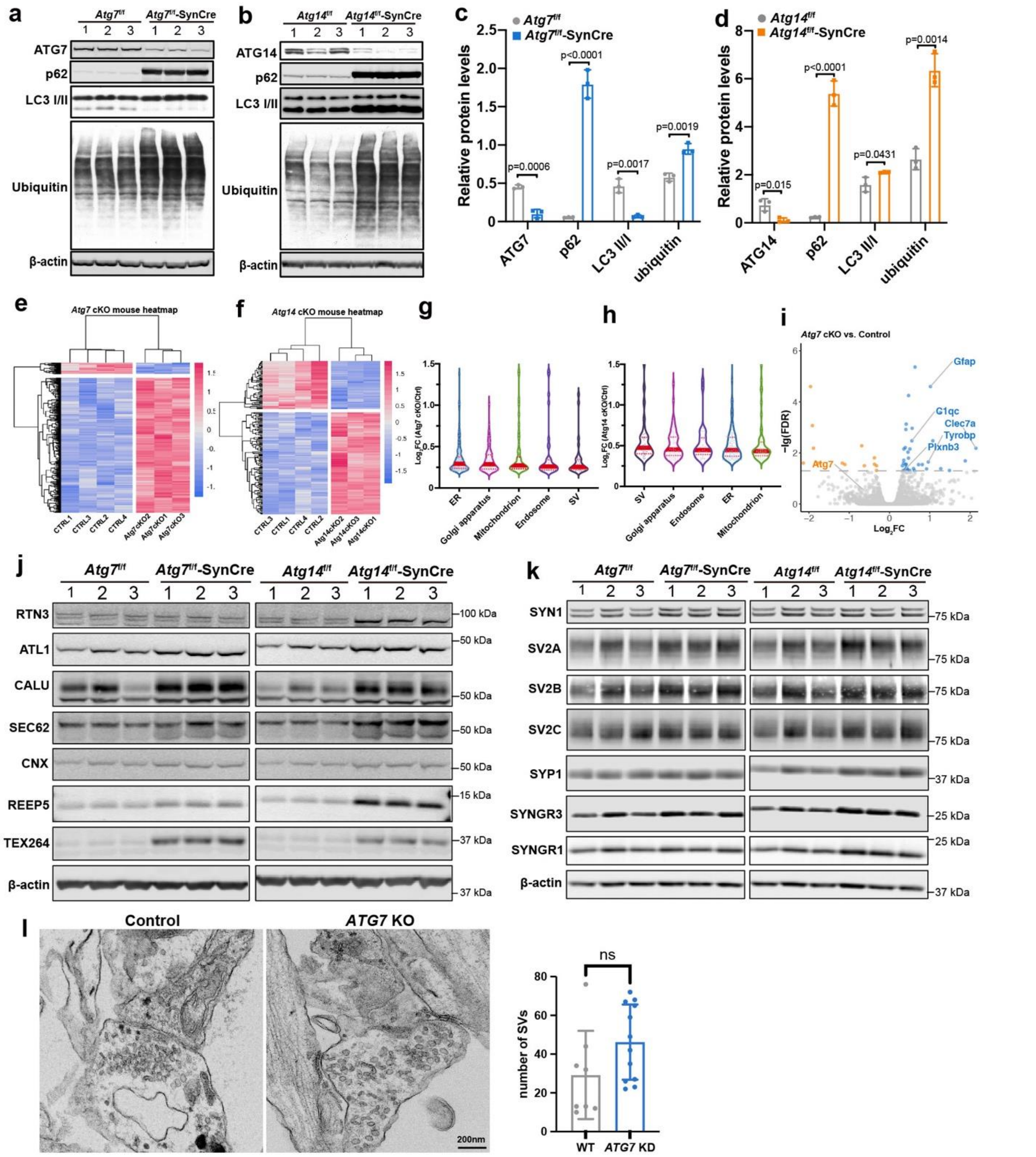

Supplementary Fig. 3. Proteomic and transcriptomic analyses of *Atg7* or *Atg14* cKO mouse brains.

(a-b) Immunoblot analysis of indicated autophagy marker proteins in *Atg7*$^{f/f}$ and *Atg7*$^{f/f}$-SynCre (a); *Atg14*$^{f/f}$ and *Atg14*$^{f/f}$-SynCre (b) mouse brains (2-months old), n= 3.

(c-d) Quantification of the change of indicated autophagy marker proteins from (a) and (b). Relative protein levels were normalized to the loading control β-actin. N=3 mice, two-sided unpaired *t*-test. All data are shown as mean ± SEM.

(e-f) Heatmap analysis of Log_2FC of DEPs ($p < 0.05$, $| Log_2FC | > 2SD$) for *Atg7* cKO (e) and *Atg14* cKO (f) mouse proteomics samples.
(g-h) Violin plots of DEPs in ER, SV, Golgi apparatus, mitochondria, and endosome based on the GO analysis in (Figure 3e) and (Figure 3f), respectively. Each dot represents one protein. Solid red bars indicate the median Log_2FC, and the red dashed bars specify the 25th and 75th interquartile range.
(i) Volcano plot of the DEGs from transcriptomic data of *Atg7*$^{f/f}$ and *Atg7*$^{f/f}$-SynCre mouse brains (n=3). Blue dots represent upregulated genes (FDR< 0.05, $Log_2FC > 0$) and orange dots represent downregulated genes. Dashed line is at -Lg (FDR)= 1.3.
(j) Immunoblot analysis of ER proteins as indicated in *Atg7*$^{f/f}$ and *Atg7*$^{f/f}$-SynCre (left); *Atg14*$^{f/f}$ and *Atg14*$^{f/f}$-SynCre (right) mouse brains (2-month-old) (n= 3).
(k) Immunoblot analysis of SV proteins as indicated in *Atg7*$^{f/f}$ and *Atg7*$^{f/f}$-SynCre (left); *Atg14*$^{f/f}$ and *Atg14*$^{f/f}$-SynCre (right) mouse brains (2-month-old) (n= 3).
(l) Electron microscopy (EM) images of synapse and synaptic vesicles (SV) Control (n=8) and *ATG7* KD (n=12) iNeurons (left). Scale bar, 200 nm. Quantification of SV numbers in the presynapse area. two-sided unpaired *t*-test. Ns, no significance. All data are shown as mean ± SEM.

Supplementary Fig. 4

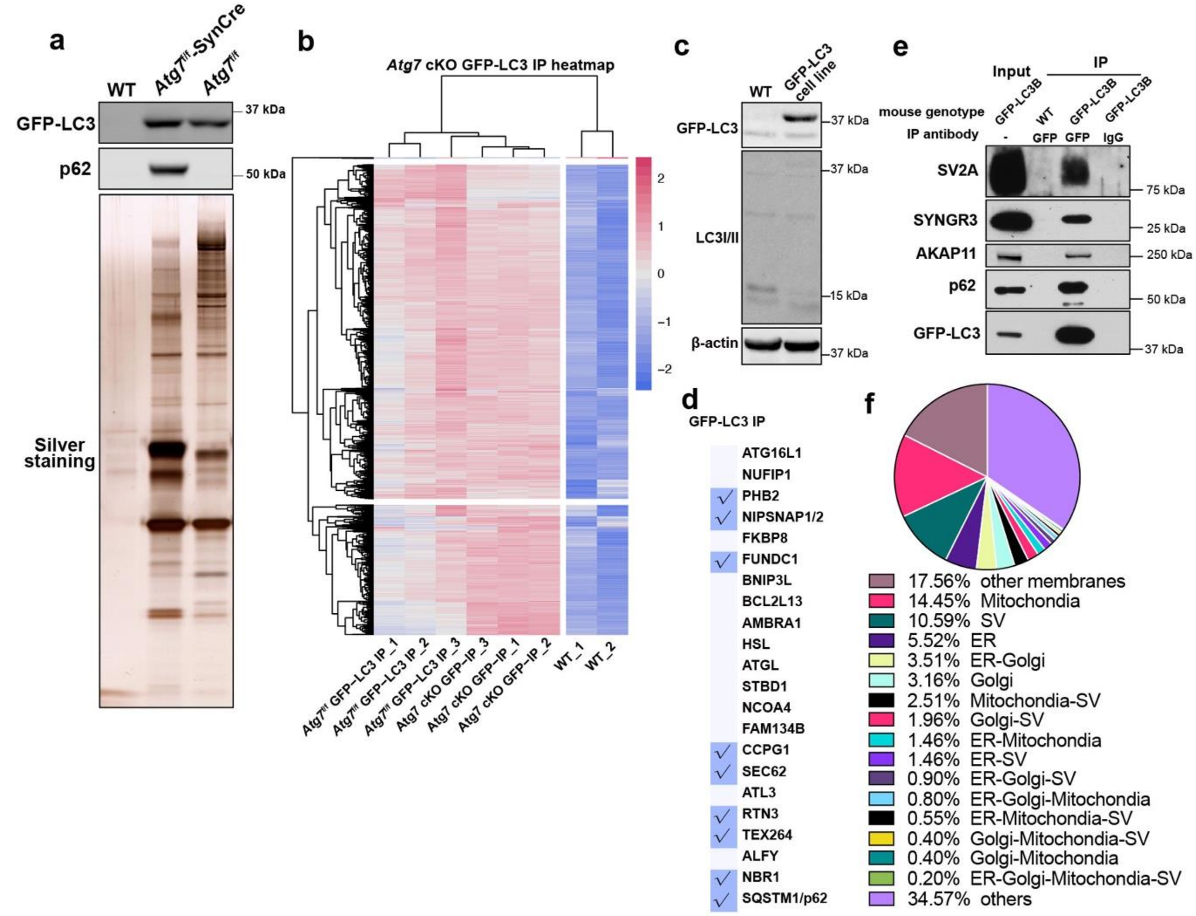

Supplementary Fig. 4. Isolation and detection of LC3-interacting proteins from mouse brains

(a) Protein chemistry study of indicated proteins for *GFP-LC3*; *Atg7*$^{f/f}$ or *GFP-LC3*; *Atg7*$^{f/f}$-SynCre mouse brain samples subjected to GFP-LC3 affinity purification and subsequent proteomics. Top and middle panels, immunoblot analysis with indicated antibodies. Bottom, silver staining of proteins after GFP-LC3 affinity purification.

(b) Heatmap analysis of Log_2FC of DEPs ($p < 0.05$, | Log_2FC |>2SD, SD = 0.21) for samples from GFP-LC3 affinity purification proteomics.

(c) Immunoblot analysis confirms the endogenous LC3 and GFP-LC3 proteins in the human WTC11 iPSC cells line stably expressing GFP-LC3.

(d) A table of the known autophagy receptors and those detected (check marks) in proteomic analysis of *Atg7*$^{f/f}$-SynCre; GFP-LC3 IP ($p < 0.05$, Log_2FC >2SD, SD = 0.21). The list of autophagy receptors was manually collated through literature searches.

(e) Pie chart analysis of the percentage of proteins categorized into different organelles or pathways from upregulated DEPs identified *GFP-LC3*; *Atg7* cKO IP compared to the WT control ($p < 0.05$, Log_2FC >2SD, SD = 0.21). Categories were summarized from GO term analysis.

Supplementary Fig. 5

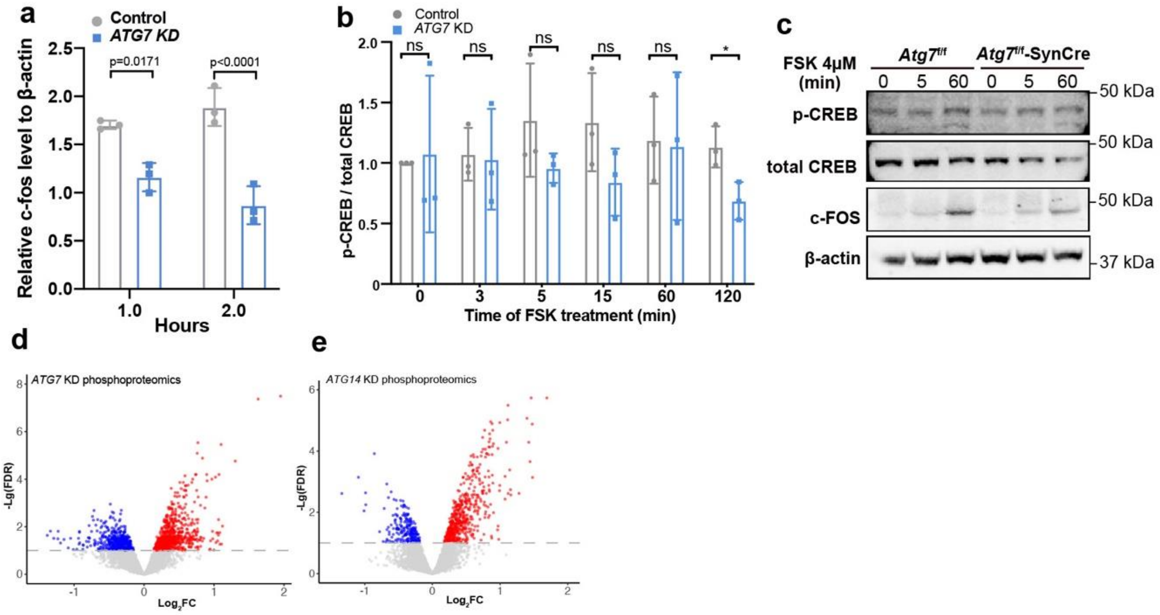

Supplementary Fig. 5. Analysis of PKA kinase activity in *ATG7* KD iNeurons and mouse primary neurons from *Atg7* cKO mice.

(a) Quantification of p-CREB normalized to total CREB in i3N-derived control and *ATG7* KD human iNeurons treated with Forskolin (4 μM) for 3, 5, 15, 60, 120 minutes in figure (5e). 3 biologically independent replicates, two-sided unpaired *t*-test.

(b) Quantification of c-FOS in i3N-derived control and *ATG7* KD human iNeurons treated with Forskolin (4 μM) for 60, 120 minutes in figure (5e). Relative protein levels were normalized to β-actin. Data were collected from 3 biologically independent replicates, two-sided unpaired *t*-test. ns, no significance.

(c) Immunoblot analyses of indicated proteins in primary cortical neurons (DIV14) cultured from *Atg7*$^{f/f}$ and *Atg7*$^{f/f}$-SynCre mice after time-dependent (baseline, 5 minutes, 1 h) Forskolin treatment (4 μM). Representative images from two independent replicates.

(d-e) Volcano plots of differentially phosphorylated proteins identified from phosphoproteomic analyses in *ATG7* KD (left) and *ATG14* KD (right) iNeurons. Red and blue dots represent proteins with an increased phosphorylation (FDR< 0.05, Log_2FC> 0) and decreased phosphorylation (FDR< 0.05), respectively. Dashed line is at -Lg (FDR)= 1.3.

All data are shown as mean ± SEM.
