# Supplementary material for: Integrated proteomics reveals autophagy landscape and an autophagy receptor controlling PKA-RI complex homeostasis in neurons
Source: Nat Commun. 2024 Apr 10;15:3113. doi: 10.1038/s41467-024-47440-z (PMC11006854; doi:10.1038/s41467-024-47440-z)
Supplement: Supplementary file 3 — Description of Additional Supplementary Files [file 41467_2024_47440_MOESM3_ESM.pdf]

Description of Additional Supplementary Files

Supplementary Data 1A. Summary of whole and phosphor proteomics sample information in this study.

Supplementary Data 1B. The cells were lysed, digested, labeled by TMT reagents and analyzed by TMT-LC/LC-MS/MS. Gene names: extracted from Swissprot and UCSC database; protein accession: from these two databases; PSM#: the total number of peptide-spectrum matches for each protein; peptide#: the total number of peptides identified for each protein; coverage (%): the percentage of the amino acid sequence in a protein that is sequenced. Differentially expressed (DE) proteins between WT and other genotypes were analyzed by the moderated t-test in LIMMA (R package). Out of 7,991 proteins, there are 1,460 and 1,172 DE proteins in ATG7 KO and ATG14 KO iPSC, respectively (highlighted in different colors, $p < 0.05$, |log2ratio| > 2-fold SD, SD = 0.18). *Final FDR values were below 0.1 based on premutation analysis (Tan et al. Immunity. 2017, 46(3), 488-503.).*

Supplementary Data 1C. The mouse brain samples were lysed, digested, labeled by TMT reagents and analyzed by TMT-LC/LC-MS/MS. Gene names: extracted from Swissprot and UCSC database; protein accession: from these two databases; PSM#: the total number of peptide-spectrum matches for each protein; peptide#: the total number of peptides identified for each protein; coverage (%): the percentage of the amino acid sequence in a protein that is sequenced. Differentially expressed (DE) proteins between WT and other genotypes were analyzed by the moderated t-test in LIMMA (R package). Out of 9,638 proteins, there are 587 DE proteins in neuron-specific *Atg7* KO mice (highlighted in different colors, $p < 0.05$, |log2ratio| > 2-fold SD, SD = 0.1). *Final FDR values were below 0.1 based on premutation analysis (Tan et al. Immunity. 2017, 46(3), 488-503.).*

Supplementary Data 1D. The mouse brain samples were lysed, digested, labeled by TMT reagents and analyzed by TMT-LC/LC-MS/MS. Gene names: extracted from Swissprot and UCSC database; protein accession: from these two databases; PSM#: the total number of peptide-spectrum matches for each protein; peptide#: the total number of peptides identified for each protein; coverage (%): the percentage of the amino acid sequence in a protein that is sequenced. Differentially expressed (DE) proteins between WT and KO were analyzed by the moderated t-test in LIMMA (R package). Out of 6,252 proteins, there are 591 DE proteins in neuron-specific *Atg14* KO mice (highlighted in different colors, $p < 0.05$, |log2ratio| > 2-fold SD, SD = 0.16). *Final FDR values were below 0.1 based on premutation analysis (Tan et al. Immunity. 2017, 46(3), 488-503.).*

Supplementary Data 1E. LC3 and its interacted proteins were purified from GFP-LC3 overexpressed Atg7f/f-Syn-Cre mouse brains. The proteins were then digested, labeled by TMT reagents and analyzed by TMT-LC/LC-MS/MS. Gene names: extracted from

Swissprot and UCSC database; protein accession: from these two databases; PSM#: the total number of peptide-spectrum matches for each protein; peptide#: the total number of peptides identified for each protein. Differentially expressed (DE) proteins between WT and other genotypes were analyzed by the moderated t-test in LIMMA (R package), followed by false discovery rate (FDR) analysis by the Benjamini-Hochberg method. Out of 2,147 proteins, there are 1980 DE proteins in cytoplasm ($FDR < 0.05$, $|log2ratio| > 2$-fold SD, SD = 0.21) in neuron-specific Atg7 KO mice, (highlighted in different colors).

Supplementary Data 1F. Phosphoproteomics of human ATG7 and ATG14 knockout in human pluripotent stem cell induced neurons.
