# Supplementary material for: Integrated proteomics reveals autophagy landscape and an autophagy receptor controlling PKA-RI complex homeostasis in neurons
Source: Nat Commun. 2024 Apr 10;15:3113. doi: 10.1038/s41467-024-47440-z (PMC11006854; doi:10.1038/s41467-024-47440-z)
Supplement: Supplementary file 5 — Reporting Summary [file 41467_2024_47440_MOESM5_ESM.pdf]

nature portfolio

Corresponding author(s): Zhenyu Yue

Last updated by author(s): Mar 1, 2024

Reporting Summary

Nature Portfolio wishes to improve the reproducibility of the work that we publish. This form provides structure for consistency and transparency in reporting. For further information on Nature Portfolio policies, see our Editorial Policies and the Editorial Policy Checklist.

Statistics

For all statistical analyses, confirm that the following items are present in the figure legend, table legend, main text, or Methods section.

n/a	Confirmed	
☐	☒	The exact sample size (*n*) for each experimental group/condition, given as a discrete number and unit of measurement
☐	☒	A statement on whether measurements were taken from distinct samples or whether the same sample was measured repeatedly
☐	☒	The statistical test(s) used AND whether they are one- or two-sided<br>*Only common tests should be described solely by name; describe more complex techniques in the Methods section.*
☐	☒	A description of all covariates tested
☐	☒	A description of any assumptions or corrections, such as tests of normality and adjustment for multiple comparisons
☐	☒	A full description of the statistical parameters including central tendency (e.g. means) or other basic estimates (e.g. regression coefficient) AND variation (e.g. standard deviation) or associated estimates of uncertainty (e.g. confidence intervals)
☐	☒	For null hypothesis testing, the test statistic (e.g. *F*, *t*, *r*) with confidence intervals, effect sizes, degrees of freedom and *P* value noted<br>*Give P values as exact values whenever suitable.*
☐	☒	For Bayesian analysis, information on the choice of priors and Markov chain Monte Carlo settings
☐	☒	For hierarchical and complex designs, identification of the appropriate level for tests and full reporting of outcomes
☐	☒	Estimates of effect sizes (e.g. Cohen's *d*, Pearson's *r*), indicating how they were calculated

Our web collection on statistics for biologists contains articles on many of the points above.

Software and code

Policy information about availability of computer code

Data collection	Imaging : Zen 2011 software (v2.6)
Data analysis	Fiji (ImageJ for image analysis), GraphPad Prism v10 (for quantification and statistical analysis)

For manuscripts utilizing custom algorithms or software that are central to the research but not yet described in published literature, software must be made available to editors and reviewers. We strongly encourage code deposition in a community repository (e.g. GitHub). See the Nature Portfolio guidelines for submitting code & software for further information.

Data

Policy information about availability of data

All manuscripts must include a data availability statement. This statement should provide the following information, where applicable:

- Accession codes, unique identifiers, or web links for publicly available datasets
- A description of any restrictions on data availability
- For clinical datasets or third party data, please ensure that the statement adheres to our policy

The authors declare that all data supporting the findings of this study are available within the paper and its supplementary information files.

Research involving human participants, their data, or biological material

Policy information about studies with human participants or human data. See also policy information about sex, gender (identity/presentation), and sexual orientation and race, ethnicity and racism.

Reporting on sex and gender	Not applicable, no human participants or human data were included in this study.
Reporting on race, ethnicity, or other socially relevant groupings	*Please specify the socially constructed or socially relevant categorization variable(s) used in your manuscript and explain why they were used. Please note that such variables should not be used as proxies for other socially constructed/relevant variables (for example, race or ethnicity should not be used as a proxy for socioeconomic status).*<br>*Provide clear definitions of the relevant terms used, how they were provided (by the participants/respondents, the researchers, or third parties), and the method(s) used to classify people into the different categories (e.g. self-report, census or administrative data, social media data, etc.)*<br>*Please provide details about how you controlled for confounding variables in your analyses.*
Population characteristics	*Describe the covariate-relevant population characteristics of the human research participants (e.g. age, genotypic information, past and current diagnosis and treatment categories). If you filled out the behavioural & social sciences study design questions and have nothing to add here, write "See above."*
Recruitment	*Describe how participants were recruited. Outline any potential self-selection bias or other biases that may be present and how these are likely to impact results.*
Ethics oversight	*Identify the organization(s) that approved the study protocol.*

Note that full information on the approval of the study protocol must also be provided in the manuscript.

Field-specific reporting

Please select the one below that is the best fit for your research. If you are not sure, read the appropriate sections before making your selection.

☒ Life sciences ☐ Behavioural & social sciences ☐ Ecological, evolutionary & environmental sciences

For a reference copy of the document with all sections, see nature.com/documents/nr-reporting-summary-flat.pdf

Life sciences study design

All studies must disclose on these points even when the disclosure is negative.

Sample size	No statistical method was used in deciding sample sizes. The sample size per group was determined from previous publications with similar methodologies (Insup et al., 2020, Nature Communications, for animal numbers).
Data exclusions	No data were excluded.
Replication	Replication of experiments was successful in three different experiments/cohort and data is accumulated during the analysis process. Results also were replicated in independent experimental setting by different authors.
Randomization	All cells analyzed for the quantification were randomly selected. Control mice and their littermate mutant/KO mice were collected from each cohort depending on the genotype and used for the experiment.
Blinding	Cell counting in human iNeurons and mouse brain immunofluoscence staining experiments were confirmed by a blinded test. Also, cell assay experiments were performed by more than two people in a blinded test. Protein analyses using Western blotting were not performed blind to load samples by order.

Reporting for specific materials, systems and methods

We require information from authors about some types of materials, experimental systems and methods used in many studies. Here, indicate whether each material, system or method listed is relevant to your study. If you are not sure if a list item applies to your research, read the appropriate section before selecting a response.

Materials & experimental systems

n/a	Involved in the study
☐	☒ Antibodies
☐	☒ Eukaryotic cell lines
☒	☐ Palaeontology and archaeology
☐	☒ Animals and other organisms
☒	☐ Clinical data
☒	☐ Dual use research of concern
☒	☐ Plants

Methods

n/a	Involved in the study
☒	☐ ChIP-seq
☒	☐ Flow cytometry
☒	☐ MRI-based neuroimaging

Antibodies

Antibodies used

SEC62 (Abcam, #ab140644, 1:1000), ATL1 (Cell Signaling Technology, #12728, 1:1000), RTN3 (Proteintech, # 12055-2-AP, 1:1000), REEP5 (Proteintech, #14643, 1:5000), Calnexin (Cell Signaling Technology, #2679P, 1:1000), Calnexin (Invitrogen, #MA3-027, 1:1000), TEX264 (NOVUS biologicals, #NBP1-89866, 1:1000), SV2A (SYSY, #119022, 1:1000), SV2B (SYSY, #119102, 1:1000), SV2C (SYSY, #119202, 1:1000), SYNGR3 (santa cruz, # sc-271046, 1:5000), SYNGR1 (SYSY, #103002, 1:1000), SYP (SYSY, #101011, 1:1000), synapsin1 (synaptic systems, #106004, 1:1000), AKAP11 (Life Span Bio Sciences, #LS-C374339-200, 1:1000), PKA RIα (Cell Signal, #5675, 1:1000), PKA RIIα (BD Bio Sciences, #612242, 1:1000), Cα (Cell Signal, #4782S, 1:1000), NeuN (Sigma-Aldrich, #MAB377, 1:1000), ubiquitin (DAKO, #Z0458, 1:1000), c-FOS (Abcam, #AB208942, 1:1000), c-FOS (SYSY, #226017, 1:1000), CREB (Abcam, #ab31387, 1:1000), p-CREB Ser133 (Cell Signal, #9198S, 1:1000), MAP2 (Abcam, #ab5392, 1:1000), MAP2 (Sigma-Aldrich, #M4403, 1:1000), ATG7 (Cell Signaling Technology, #8558S, 1:1000), ATG14 (MBL, #PD026-006, 1:1000), p62 (MBL, #PM066, 1:1000), GAPDH (Invitrogen, #MA5-15738, 1:1000), b-actin (Cell Signaling Technology, #3700S, 1:1000), HSP90 (Cell Signaling, #4874S, 1:1000), LC3B (IF) (Cell Signaling Technology, #3868S, 1:200), LC3A/B (WB) (Cell Signaling Technology, #12741S, 1:1000), GFP (Abcam, #ab13970, 1:1000), GFP (Thermofisher, #A11122, 1:1000), mouse-HRP (Jackson ImmunoResearch, #715-035-150, 1:10000), rabbit-HRP (Jackson ImmunoResearch, #711-035-152, 1:10000), Rat-HRP (Santa Cruz Biotechnology, #sc2006, 1:1000), IRDye® 800CW Donkey anti-Rabbit IgG (Licor, #926-32213, 1:10000), IRDye® 800CW Donkey anti-Mouse IgG (Licor, #926-32212, 1:10000), IRDye® 680RD Donkey anti-Rabbit IgG (Licor, #926-68072, 1:10000), IRDye® 680RD Donkey anti-Mouse IgG (Licor, #926-68073, 1:10000), IRDye® 800CW Donkey anti-Guinea Pig IgG (Licor, #926-32411, 1:10000), IRDye® 800CW Goat anti-Rat IgG (Licor, #926-32219, 1:10000), Alexa Fluor 555 donkey anti-rabbit IgG (Invitrogen, #A32794, 1:500), Alexa Fluor 647 goat anti-rabbit IgG (Invitrogen, #A21244, 1:500), Alexa Fluor 488 donkey anti-mouse IgG (Invitrogen, #A32766, 1:500), Alexa Fluor 647 donkey anti-mouse IgG (Invitrogen, #A31571, 1:500), Alexa Fluor 488 goat anti-chicken IgG (Invitrogen, #A11039, 1:500), Alexa Fluor 647 donkey anti-chicken IgG (Invitrogen, #A21449, 1:500), Alexa Fluor 488 donkey anti-rat IgG (Invitrogen, #A21208, 1:500).

Validation

primary antibodies are calidated by the source companies (listed in Methods section), previous use in unrelated published studies, as well as confirmed by us via recognition of a correctly-sized band on immunoblots or correct protein expression pattern on immunofluorescence staining.

Eukaryotic cell lines

Policy information about cell lines and Sex and Gender in Research

Cell line source(s)

Human H1 stem cell line(a male cell line), i3N cell line(a male cell line), MONO-ALLELIC mEGFP-TAGGED MAP1LC3B WTC iPSC LINE(a male cell line), HEK293T(a female cell line)

Authentication

The cell lines used in this study was fully authenticated by the vendor, ATCC (VA, USA).

STR Profile for HEK293T (https://www.atcc.org/Products/All/CRL-3216.aspx#specifications)
- CSF1PO: 11,12
- D13S317: 12,14
- D16S539: 9,13
- D5S818: 8,9
- D7S820: 11
- TH01: 7, 9.3
- TPOX: 11
- vWA: 16,19
- Amelogenin: X

STR Profile for human H1 stam cell line:
- D16s539: 5, 8-15
- D7S820: 6-14
- D13S317: 7-15
- D5S818: 7-15
- CSF1PO: 6-15
- TPOX: 6-15
- Amelogenin: NA
- TH01: 5-11
- vWA: 11, 13-21

STR profile for MONO-ALLELIC mEGFP-TAGGED MAP1LC3B WTC iPSC Line was confirmed in 29 allelic polymorphisms across 15 STR loci compared to donor fibroblasts and the karyotype was normal
The inducible i3N iPS cell line is a gift from Dr. Martin Kampmann's lab, which was derived from WTC11 parental cell line.The

	cell line has been confirmed to have a normal karyotype.
Mycoplasma contamination	Mycoplasma tested was performed every month to confirm the cell line was mycoplasma negative.
Commonly misidentified lines (See ICLAC register)	No

Animals and other research organisms

Policy information about studies involving animals; ARRIVE guidelines recommended for reporting animal research, and Sex and Gender in Research

Laboratory animals	Atg7flox/flox mice mice were kindly gifted from Dr. Masaaki Komatsu (Tokyo, Japan).<br>Atg14flox/flox mice were kindly gifted from Dr. Herbert W.Virgin (Washington University School of Medicine, St. Louis, MO).<br>2-6months-old mice were used for breeding and 1month-old mice were used for genotyping.<br>Mice were maintained in rooms with 12 light/12 dark cycle, 23°C, 40-60% humidity, and water accessible at all times.
Wild animals	No wild animals were used in this study.
Reporting on sex	Male and female mice were included at approximately 1:1 ratio.
Field-collected samples	No field-collected samples were used in this study.
Ethics oversight	All experiments conducted in this study comply with all relevant ethical regulations. All animal experiments were approved by the Institutional Animal Care and Use Committee (IACUC) at Icahn school of Medicine at Mount Sinai.

Note that full information on the approval of the study protocol must also be provided in the manuscript.

Plants

Seed stocks	No seed stocks were included in this study.
Novel plant genotypes	No novel plants were included in this study.
Authentication	Not applicable.
